# Analgesic impact of buprenorphine transdermal patch in total hip arthroplasty: A randomized controlled trial protocol

**DOI:** 10.1097/MD.0000000000020405

**Published:** 2020-06-12

**Authors:** Wen-Min Li, Feng-Dao Li, Hua Xu, Li-Chen Sun

**Affiliations:** a Department of Medicine, Linyi Cancer Hospital; b Department of Orthopedics, Yinan County Hospital of Traditional Chinese Medicine; c Department of Orthopedics, Linyi Cancer Hospital, Shandong, China.

**Keywords:** buprenorphine transdermal patch, pain, randomized controlled trial, study protocol, total hip arthroplasty

## Abstract

**Background:**

The efficacy and safety of buprenorphine transdermal patch (BTP) has been well established in chronic pain, but data regarding acute postoperative pain relief is still very limited. Therefore, we design a prospective, randomized, controlled study to evaluate the effectiveness and safety of the BTP for postoperative analgesia in total hip arthroplasty.

**Methods:**

This study is designed as a single-center, prospective, double-blind, randomized controlled trial. Group A receives a 10 mg patch of buprenorphine at the conclusion of surgery which is continued for 14 days. Group B receives a conventional analgesic regimen, that is, IV paracetamol 1 mg every 8 hours alternating with parenteral tramadol 50 mg every 8 hours for the first 2 postoperative days followed by oral administration of the same drug still the end of 2 weeks. A total of 160 patients are needed with an allowance for 10% drop-out. The primary outcome of this noninferiority study is opioid consumption within the first 24 hours following surgery. The secondary outcomes included numerical rating scale scores at rest, postoperative complications, length of hospital stay, and patient satisfaction.

**Results:**

This trial is expected to be the largest randomized trial assessing the efficacy of BTP after primary total hip arthroplasty and powered to detect a potential difference in the primary outcome.

**Trial registration number:**

This study protocol was registered in Research Registry (researchregistry5524).

## 1 Introduction

Total hip arthroplasty (THA) has been one of the most successful medical procedures from the start, alleviating pain and restoring function in patients suffering from the consequences of a spectrum of hip pathologies.^[1]^ However, THA causes moderate to severe postoperative pain, and inadequate perioperative analgesia management delays ambulation and decreases the quality of postoperative recovery. To help control pain, patients can receive a multimodal drug regimen, ice, and physical therapy.^[2]^

Buprenorphine is a semi-synthetic opioid, a derivative of the opium alkaloid thebaine. Buprenorphine has been in regular clinical use for 3 decades, administered as sublingual tablets or as injections for acute or chronic pain.^[3]^ Pharmacodynamic and pharmacokinetic properties of buprenorphine have recently been thoroughly updated and documented in a review by Kress.^[4]^ The longer half-life of buprenorphine and its high affinity to the μ-receptor, make it longer lasting and 25 to 100 times more efficacious (per mg) as an analgesic than morphine.

The molecular structure of buprenorphine confers suitability for use in a variety of preparations, including the transdermal route. Transdermal drug delivery systems are non-invasive, simple, compliant, and sustained methods of delivery. Buprenorphine is highly lipophilic and therefore suitable for transdermal administration. For usage as transdermal patch, buprenorphine is incorporated into an adhesive polymer matrix (acrylate vinyl acetate), from which it is continuously released into the systemic circulation over a period of 7 days. Buprenorphine transdermal patch (BTP) is available in 3 strengths 5, 10, and 20 mg with rate of drug release of 5, 10, and 20 μg/h, respectively.^[5]^ Following removal of the patch, concentrations decrease to about one-half in 12 hours, and then decline more gradually with an apparent half-life of about 26 hours. These pharmacokinetics should minimize some of the adverse effects typical of fast onset, fast offset delivery of potent opioids. It allows a practical means of administering the drug to patients who may be unable to take medication orally and to elderly patients with cognitive dysfunction or impaired memory.^[6]^

As far as we know, the efficacy and safety of BTP has been well established in chronic pain, but data regarding acute postoperative pain relief is still very limited. In addition, few studies have been performed on the use of the BTP for THA surgery.^[3]^ Therefore, we design a randomized controlled trial (RCT) to evaluate the effectiveness and safety of the BTP for postoperative analgesia in THA.

## 2 Materials and method

### 2.1 Trial design

This study is designed as a single-center, prospective, double-blind RCT. The participants will be randomly assigned to either the BTP group or the control group.

### 2.2 Ethics

This study was approved by the institutional review board in our hospital (B200410023) and was registered in Research Registry (researchregistry5524). This trial will be performed in accordance with the principles of the Declaration of Helsinki (Edinburgh 2000 version). Written informed consent will be obtained from each participant before enrollment.

### 2.3 Randomization and blinding

A pharmacist will provide a set of 160 random numbers for the allocation sequence using a website (http://www.randomization.com). None of the investigators, except for the pharmacists, will be aware of the block size. The random allocation sequence will be available to pharmacists only and thus it will be concealed from the other research team members. The included participants will be randomly assigned to either the BTP group or the control group in a ratio of 1:1. The pharmacist who administers the drugs has no further role in the study; the surgeons, anesthesiologists, and nurses providing intra- and postoperative care, as well as the research coordinator assessing outcomes, are all kept blinded to allocation results.

### 2.4 Eligibility criteria

All patients over the age of 18 years, who are scheduled for a primary unilateral THA for osteoarthritis or avascular necrosis between May 2020 and July 2021, are eligible for inclusion in the study. Patients with revision surgery, bilateral surgery, previous enrolment in the study are excluded. Patients unable to give consent or with any known contraindications to medications, regional, or neuraxial anesthesia are excluded. Patients with daily intake of strong opioids (morphine, methadone, fentanyl, hydromorphone), a history of intravenous drug abuse and alcohol abuse are also excluded from the study. Participants are informed that the study is evaluating the efficacy and safety of BTP for pain control following primary THA and that they are randomly assigned to either the BTP and control group.

### 2.5 Interventions

Group A receives a 10 mg patch of buprenorphine at the conclusion of surgery which is continued for 14 days. Group B receives a conventional analgesic regimen, that is, IV paracetamol 1 mg every 8 hours alternating with parenteral tramadol 50 mg every 8 hours for the first 2 postoperative days followed by oral administration of the same drug still the end of 2 weeks. No other opioid medications, non-steroidal anti-inflammatory drugs (other than diclofenac for rescue analgesia), or antidepressants are used.

### 2.6 Surgery and rehabilitation

All patients are managed with general anesthesia without regional block or epidural anesthesia. No periarticular injection of local anesthetic is used in any of the study patients. All surgeries are performed by 1 of the 2 surgeons. In all cases, a minimally invasive anterolateral approach is used with the patients placed in a lateral decubitus position. We use a cementless cup in all cases for the acetabular component. All patients begin gait exercise and range of motion training without any precautions on the day after surgery.

### 2.7 Outcome measurements

#### 2.7.1 Primary outcome measure

The primary outcome of this noninferiority study is opioid consumption within the first 24 hours following surgery. Subjects receive standardized postoperative multimodal analgesics. Supplemental oxycodone 5 to 15 mg every 3 hours for pain is available to each subject. Nurses administer a 5 mg oxycodone tablet for numerical rating scale (NRS) scores of 1 to 3, 10 mg for NRS scores of 4 to 6, and 15 mg for NRS scores of 7 to 10. Oral hydromorphone is substituted for oxycodone in the instances of patient allergy or intolerance. Rescue analgesia is available with IV hydromorphone 0.5 mg every 1 hour as needed for pain of greater than NRS of 7 when refractory to oral opioids. Opioid consumption during the first 48 hours postoperatively is retrieved from the electronic medical record and convert to IV morphine equivalents for analysis. The average and worst NRS pain scores at rest and with activity are determined by blinded investigators at 24 and 48 hours postoperatively.

#### 2.7.2 Secondary outcome measures

The secondary outcomes included NRS scores at rest, postoperative complications, length of hospital stay, and patient satisfaction. The NRS scores at rest are measured at 12, 24, 48, and 72 hours postoperatively. Any postoperative complications occurring during the course of the trial are recorded. Length of hospital stay is calculated by measuring the time from the completion of surgery through discharge for each patient. Subjects are asked to give a verbal assessment representative of the quality of analgesia at 24 and 48 hours postoperatively. Response to this assessment is recorded as “satisfied”’ or “unsatisfied.”

### 2.8 Sample size calculation

We estimate that with 64 participants in each group, the study will have more than 80% power to detect a clinically important difference between the groups in regard to the change in the pain score evaluated with the NRS. This is assuming a mean intergroup difference in score of 20 mm based on previous literature and a pooled standard deviation of 35 mm on the basis of preliminary data at an alpha level of 5%. Based on this estimation, a total of 160 patients are needed with an allowance for 10% drop-out.

### 2.9 Statistical analysis

All statistics are performed using STATA software (StataCorp). Student *t* test is used to compare the primary outcome between groups in this study. For missing primary outcome data, NRS scores are replaced with the median scores for other patients of the same treatment group at the same period. Comparisons between the study groups are performed using Chi-squared test and Student *t* test for categorical and continuous variables, respectively. All tests are 2 sided, and *P* < .05 is considered statistically significant.

## 3 Results

**Figure 1:**
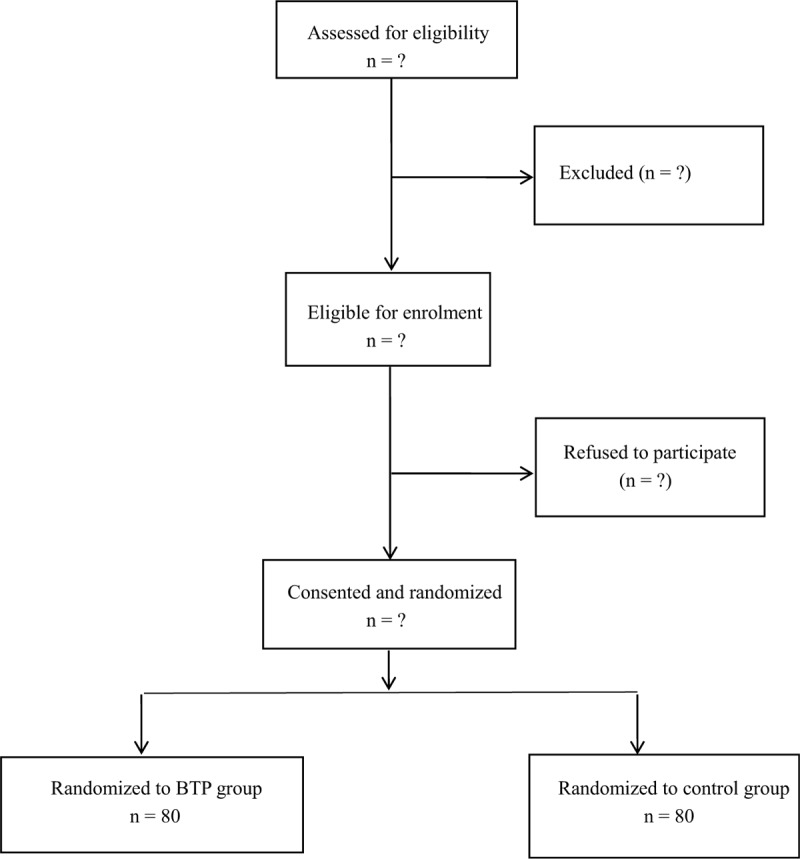
Flowchart of the trial outline.

Figure 1 shows a flowchart of the trial outline. The demographic characteristics of the patients in the 2 groups will be summarized in Table 1.

**Table 1:**
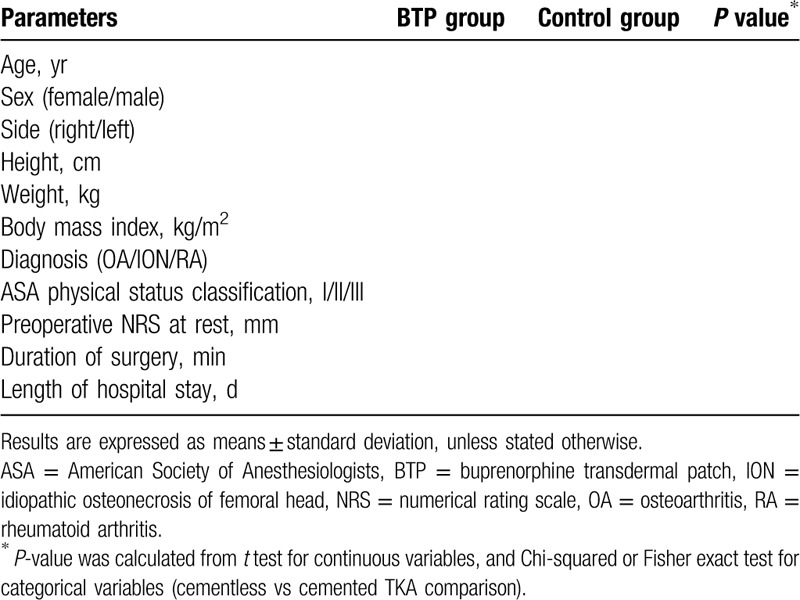
Patient demographics and baseline clinical characteristics.

## 4 Discussion

The BTP is a type of synthetic opioid analgesic, and it was licensed for the treatment of moderate-to-severe chronic pain that does not respond to nonopioid analgesics. The active ingredient of the BTP is buprenorphine, which is a potent opioid analgesic that acts primarily as a partial agonist at the μ-opioid receptor.^[7]^ The BTP offers many advantages over the typical full μ-opioid receptor agonists for treating chronic pain, and its partial agonist activity induces a ceiling effect for respiratory depression but not for analgesia, thereby resulting in a reduced risk for this potentially fatal adverse event compared with that of other full opioid agonists.^[8]^

The role of BTP in chronic malignant and non-malignant conditions is well proven. However, there are only few studies with BTP for postoperative analgesia^[9–12]^ and only 1 in THA.^[3]^ In THA surgery, inadequate postoperative pain control may cause delay in recovery, prolonged hospital stay, delay in start of physiotherapy, and may even adversely affect the outcome of surgery. Thus, the present randomized, double-blind, controlled study is undertaken to evaluate the safety and efficacy of BTP after THA.

There are several limitations of this randomized trial. First, this study does not measure levels of depression or other conditions which have been shown to influence the perception of pain. However, randomizing patients to each group decreases the likelihood of this having a significant impact on the results. Secondly, owing to the small sample size, this study is also not powered to detect a difference in length of stay or other secondary outcomes. Despite these limitations, this trial is expected to be the largest randomized trial assessing the efficacy of BTP after primary THA and powered to detect a potential difference in the primary outcome.

## Author contributions

**Conceptualization:** Li-Chen Sun.

**Data curation:** Wen-Min Li, Hua Xu.

**Formal analysis:** Wen-Min Li, Feng-Dao Li, Hua Xu.

**Funding acquisition:** Li-Chen Sun.

**Investigation:** Wen-Min Li, Feng-Dao Li, Hua Xu.

**Methodology:** Li-Chen Sun.

**Resources:** Li-Chen Sun.

**Software:** Feng-Dao Li.

**Supervision:** Li-Chen Sun.

**Validation:** Li-Chen Sun.

**Visualization:** Li-Chen Sun.

**Writing – original draft:** Wen-Min Li.

**Writing – review & editing:** Feng-Dao Li, Hua Xu.
